# 3′UTR polymorphisms of carbonic anhydrase IX determine the miR-34a targeting efficiency and prognosis of hepatocellular carcinoma

**DOI:** 10.1038/s41598-017-04732-3

**Published:** 2017-06-30

**Authors:** Kuo-Tai Hua, Yu-Fan Liu, Chia-Lang Hsu, Tsu-Yao Cheng, Ching-Yao Yang, Jeng-Shou Chang, Wei-Jiunn Lee, Michael Hsiao, Hsueh-Fen Juan, Ming-Hsien Chien, Shun-Fa Yang

**Affiliations:** 1 Graduate Institute of Toxicology, College of Medicine, National Taiwan University, Taipei, Taiwan; 2 Department of Biomedical Sciences, Chung Shan Medical University, Taichung, Taiwan; 3 Department of Medical Research, Chung Shan Medical University Hospital, Taichung, Taiwan; 4 Department of Life Science, Graduate Institute of Biomedical Electronics and Bioinformatics, National Taiwan University, Taipei, Taiwan; 5 Department of Laboratory Medicine, National Taiwan University Hospital, Taipei, Taiwan; 6 Department of Internal Medicine, National Taiwan University Hospital, Taipei, Taiwan; 7 Department of Surgery, National Taiwan University Hospital, and National Taiwan University College of Medicine, Taipei, Taiwan; 8 Genomics Research Center, Academia Sinica, Taipei, Taiwan; 9 Wan Fang Hospital, Taipei Medical University, Taipei, Taiwan; 10 Department of Urology, School of Medicine, College of Medicine, Taipei Medical University, Taipei, Taiwan; 11 Graduate Institute of Clinical Medicine, College of Medicine, Taipei Medical University, Taipei, Taiwan; 12 Institute of Medicine, Chung Shan Medical University, Taichung, Taiwan

## Abstract

Carbonic anhydrase IX (CA9) expression level has been considered as a poor prognostic factor in hepatocellular carcinoma (HCC) patients. However, the judging criteria of CA9 level is hard to define for potential clinical applications. Unlike CA9 expression level, CA9 polymorphism is poorly documented in HCC. Here, we found that people carry A allele at CA9 rs1048638, a 3′UTR SNP, has higher risk of HCC. rs1048638-CA correlates with advanced stages, larger tumor sizes, more vascular invasion, and shorter survival of HCC patients. A allele at CA9 rs1048638 impairs miR-34a, a tumor suppressor miRNA in HCC, binding to CA9 3′UTR and desensitizes CA9 mRNA to miR-34a-dependent RNA degradation. CA9 expression levels were also correlated with miR-34a levels and rs1048638 genotypes in HCC patients. rs1048638 influences HCC risk and progression through effects on miR-34a-targeted CA9 expression in HCC. In conclusion, genetic variations of the CA9 3′UTR play important roles in regulating CA9 expression and cancer progression, which is a novel determinant and target for HCC metastasis and prognosis.

## Introduction

Hepatocellular carcinoma (HCC) possesses a dismal prognosis and is the second leading cause of cancer-related deaths worldwide^1^. Surgical resection, potentially curative treatment, is only applicable to the small subset of patients diagnosed at early stages^1, 2^. Unfortunately, conventional or targeted chemotherapies have not been fully developed to have significant impacts on overall survival^3^. Therefore, it is pivotal to improve the prognosis of HCC patients by developing effective individualized treatments based on molecular classification.

Carbonic anhydrase IX (CA9) is a membrane-associated glycoprotein belonging to a family of zinc-containing enzymes. It catalyzes the reversible reaction H_2_O + CO_2_ ↔ H^+^ + HCO_3_^−^, which is crucial to tumor pH homeostasis^4, 5^. CA9 expression is induced in tumor cells under hypoxia and helps maintain a normal intracellular pH while facilitating an acidic extracellular pH^4^. An acidic extracellular microenvironment is an important feature of cancer, and also promotes cancer progression through activating proteinase activity, disrupting adherence junctions, inhibiting drug uptake, and stimulating the metastatic potential^6–8^. CA9 expression is associated with poor clinical outcomes in several tumors including head and neck, cervix, kidney, and lung cancers^9–11^. Recently, CA9 expression was also identified as a poor prognostic factor in patients with resectable HCC^12^.

**Figure 1:**
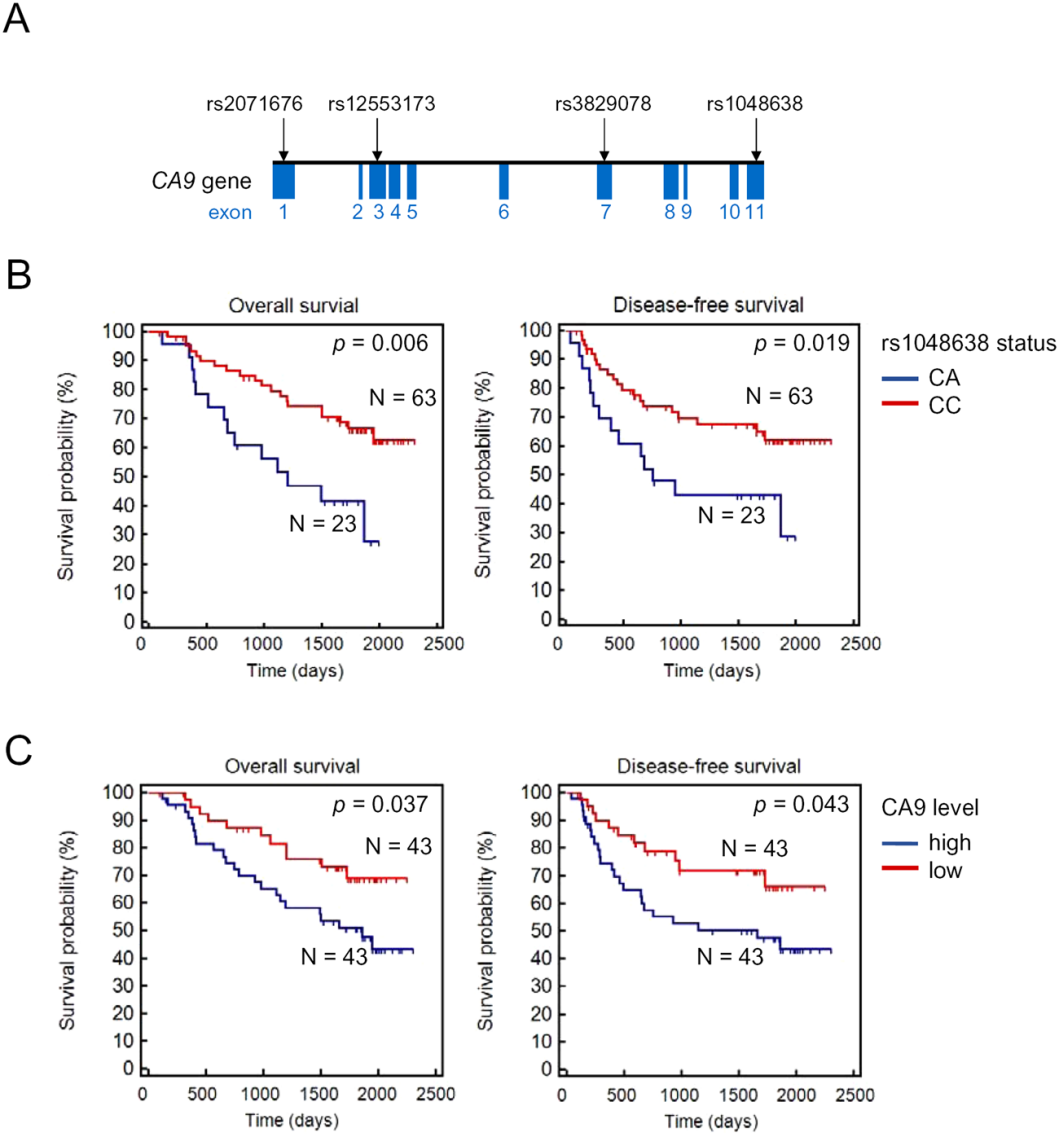
Association of rs1048638 with survival of hepatocellular carcinoma (HCC) patients. (**A**) Schematic illustration of CA9 single-nucleotide polymorphisms (SNPs) evaluate in HCC. (**B**) Kaplan-Meier analysis of the correlation between rs1048638 genotypes and overall survival (OS) and disease-free survival (DFS) of 86 HCC patients. (**C**) Kaplan-Meier analysis of the correlation between CA9 expression levels and OS or DFS of 86 HCC patients.

The human *CA9* gene is localized on chromosome 9p12-13^13^. More than 30 single-nucleotide polymorphisms (SNPs) have been identified in the *CA9* gene. Most of them are located in exon regions. These polymorphisms may affect CA9 activity and regulation, and some of them are thought to be genetic risk factors for disease susceptibility^14–16^. This suggests that genetic variations of *CA9* may also induce differences in the incidence risks and outcomes of cancers. However, little is known about the impacts of *CA9* polymorphisms on cancer susceptibility and progression of HCC. In this study, we first analyzed four SNPs (three with minor allelic frequencies of ≥5% and one reported prognostic SNP) in a cohort containing 312 HCC patients and 312 healthy volunteers (Fig. 1A). The prognostic values of one specific SNP, which is located in the *CA9* 3′untranslated region (UTR), was identified and further evaluated in another independent cohort of HCC patients (*n* = 86). To further explore the possible roles of *CA9* polymorphisms in HCC progression and metastasis, we also performed functional analyses on the selected SNP using both *in vitro* and *in vivo* assays and evaluated their correlations with CA9 expression levels in HCC. We identified a critical microRNA (miR)-34a-CA9 regulation axis controlling HCC metastasis, and CA9 polymorphisms disrupt this crucial regulation.

## Results

### The CA9 rs1048638 polymorphism increased the HCC risk

A statistical analysis of demographic characteristics of 312 HCC cases and 312 controls is shown in Table S1. There were no significant differences between cases and controls in terms of the distribution of age and sex status as a result of individual matching (*p* > 0.05). These results suggest that HCC patient data were comparable to control data. To obtain adequate power for evaluating the potential association and test the putative functional relevance of *CA9*, three SNPs, including rs2071676, rs3829078 and rs1048638 with minor allele frequencies >5% were chosen. Furthermore, another SNP of *CA9* gene (an 18-bp deletion/insertion; 376del393) was selected since this SNP was found in the cancer patients^14, 15^. For the four analyzed SNPs, the genotype distribution of controls was consistent with those expected from Hardy-Weinberg’s equilibrium (*p* > 0.05). rs1048638 was the only SNP observed to be correlated with HCC risk (Table S2). Logistic regression analyses showed that the adjusted OR for HCC for those individuals carrying heterozygotes of the rs1048638 C and A allele (rs1048638-CA) versus those with homozygotes of the rs1048638 C alleles (rs1048638-CC) was 1.769 (95% CI: 1.124~2.785). No homozygotes of rs1048638 A alleles (rs1048638-AA) were detected in HCC patients or controls.

### The CA9 rs1048638 polymorphism was correlated with a poor prognosis of HCC patients

To assess the clinical relevance of the *CA9* polymorphism, we performed a series of bivariate stratified analyses by the clinicopathologic characteristics of HCC on the selected SNPs. We observed a significant distribution difference in rs1048638 genotypes among different clinical stages, tumor sizes, and extents of vascular invasion, but not in distant metastasis, Child-Pugh grade, HBsAg, anti-HCV, or cirrhosis (Table 1). On the contrary, rs3829078 genotypes were only correlated with the surface antigen of the hepatitis B virus (HBsAg), while rs2071676 and 376del393 had no significant correlations with any clinical characteristics (Tables S3–S5). Of note, the rs1048638 polymorphism was highly correlated with vascular invasion (*p* = 0.01), the most important predictor of HCC recurrence and survival^17^. Since our primary cohort had no patient survival data, we analyzed the survival probabilities of the rs1048638 polymorphism in another independent cohort composed of 86 HCC patients with complete follow-up information. The survival analysis showed that HCC patients carrying rs1048638-CA had shorter overall (*p* = 0.006) and disease-free (*p* = 0.019) survival times (Fig. 1B), compared to those with rs1048638-CC. We also examined CA9 mRNA expression in the same cohort and divided HCC patients into high- (CA9 level above the median) and low-expression (CA9 level below the median) groups. Consistent with a recent report^12^, HCC patients with high CA9 expression also had poorer overall (OS; *p* = 0.037) and disease-free (DFS; *p* = 0.043) survival rates compared to patients with low CA9 expression (Fig. 1C). Furthermore, a univariate analysis revealed that the *CA9* rs1048638 genotype, CA9 expression level, and vascular invasion status were all significantly associated with OS (hazard ratios = 0.39, 0.48, 2.38; *p* = 0.007, 0.042, and 0.026, respectively, Table 2). A backward stepwise multivariate analysis revealed that the rs1048638 genotype was the only independent risk factor associated with OS (hazard ratio = 0.44, *p* = 0.029, Table 2). Our analysis suggested that the *CA9* rs1048638 polymorphism is an important prognostic marker for HCC patients. Considering the practical advantages of the SNP analysis, it may have better clinical potential than detecting CA9 expression levels.

**Table 1:** Clinical status and *CA9* rs1048638 genotypic frequencies in 312 patients with hepatocellular carcinoma.

Variable	CC (*N* = 255)	CA + AA (*N* = 57)	OR (95% CI)	*p* value
**Clinical Stage**				
Stage I/II	172 (67.5%)	30 (52.6%)	1.00	0.034*
Stage III/IV	83 (32.5%)	27 (47.4%)	1.865 (1.042~3.338)	
**Tumor size**				
≤T2	176 (69.0%)	30 (52.6%)	1.00	0.018*
T2	79 (31.0%)	27 (47.4%)	2.005 (1.118~3.595)	
**Vascular invasion**				
No	220 (86.3%)	39 (68.4%)	1.00	0.001*
Yes	35 (13.7%)	18 (31.6%)	2.901 (1.495~5.628)	
**Distant metastasis**				
No	243 (95.3%)	51 (89.5%)	1.00	0.088
Yes	12 (4.7%)	6 (10.5%)	2.382 (0.854~6.643)	
**Child-Pugh grade**				
A	191 (74.9%)	47 (82.5%)	1.00	0.225
B or C	64 (25.1%)	10 (17.5%)	0.635 (0.303~1.329)	
**HBsAg**				
Negative	151 (59.2%)	28 (49.1%)	1.00	0.164
Positive	104 (40.8%)	29 (50.9%)	1.504 (0.845~2.676)	
**Anti-HCV**				
Negative	132 (51.8%)	31 (54.4%)	1.00	0.720
Positive	123 (48.2%)	26 (45.6%)	0.900 (0.506~1.601)	
**Liver cirrhosis**				
Negative	53 (20.8%)	12 (21.1%)	1.00	0.984
Positive	202 (79.2%)	45 (78.9%)	0.984 (0.486~1.991)	

See also Tables S1–S5.

T2: multiple tumor of >5 cm or tumor involving a major branch of the portal or hepatic veins.

HBsAg, surface antigen of hepatitis virus; HCV, hepatitis C virus; OR, odds ratio; CI, confidence interval.

**Table 2:** Univariate and multivariate analyses of potential prognostic variables in 86 hepatocellular carcinoma patients.

Parameters	Comparison	Univariate analysis		Multivariate analysis	
		HR (95% CI)	*p*-value	HR (95% CI)	*p*-value
*CA9* rs1048638	CC; CA	0.39 (0.20 to 0.77)	0.0075*	0.44 (0.21 to 0.92)	0.029*
*CA9* level	High(>median); Low (≤median)	0.48 (0.24 to 0.97)	0.042*	0.48 (0.21 to 1.07)	0.07
Cirrhosis	Yes; No	1.36 (0.67 to 2.74)	0.393	1.35 (0.064 to 2.86)	0.436
Histological grade	G1, 2; G3, 4	1.96 (0.96 to 3.98)	0.07	1.25 (0.58 to 2.71)	0.57
Vascular invasion	Yes; No	2.38 (1.11 to 5.09)	0.026*	1.91 (0.84 to 4.33)	0.122
Viral status	HBV^+^; HBV^−^	0.82 (0.41 to 1.61)	0.563	0.66 (0.30 to 1.49)	0.729

^*^Two-sided Cox proportional hazards regression using normal approximation.

HBV, hepatitis B virus; HR, hazard ratio; CI, confidence interval.

### The CA9 rs1048638 polymorphism determined the response to miR-34a targeting

**Figure 2:**
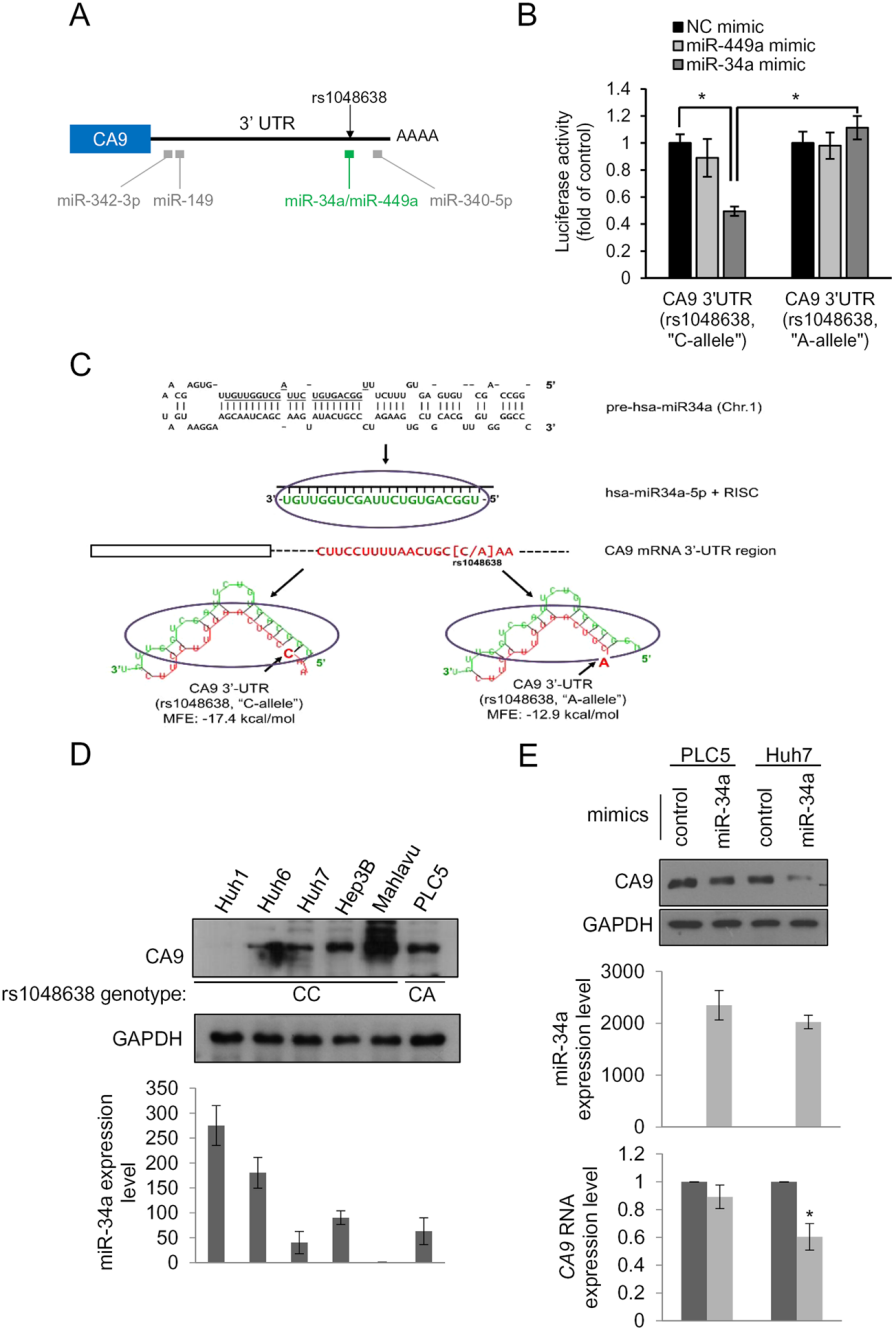
The CA9 polymorphism determines the response to miR-34a targeting. (**A**) Schematic illustration of miRNA target sites and the rs1048638 position on the CA9 3′untranslated region (UTR). (**B**) CA9 3′UTR reporters were transfected with the indicated miRNA or scrambled control (NC) mimics into 293T cells. Luciferase activities were measured and are shown as multiples of the control. (**C**) A minimum free energy (MFE) analysis of the alignment between miR-34a and the CA9 3′UTR with an “A’ or “C” allele at rs1048638. (**D**) Upper panel: Western blot analysis of CA9 in HCC cell lines. The rs1048638 genotype of each cell line is labeled below the blot. Lower panel: Quantitative analysis of miR-34a levels in HCC cell lines. Data are presented normalized to the miR-34a level of Mahlavu cells. (**E**) Upper panel: Western blot analysis of CA9 in PLC5 and Huh7 cells transfected with miR-34a or the control mimic, respectively. miR-34a expression levels are shown in the middle panel. CA9 RNA expression levels are shown in the lower panel. Three independent replicates were performed in each experiment. Error bars, ±S.D. *p < 0.05 by a two-sided Student’s *t*-test. GAPDH was used as a loading control.

Since rs1048638 is located in the 3′UTR of *CA9* mRNA, we hypothesized that a polymorphism of this SNP may affect miRNAs targeting *CA9* mRNA. To evaluate this possibility, we first analyzed the *CA9* 3′UTR in miRNA databases, including TargetScan and miRanda, for *CA9* targeting miRNAs. We found that rs1048638 was located in the target sequence of miR-34a and miR-449a (Fig. 2A). We next constructed the *CA9* 3′UTR of both rs1048638 genotypes to luciferase reporters and evaluated their activities in the presence of miR-34a or miR-449a mimics. As shown in Fig. 2B, the miR-34a but not the miR-449a mimic significantly decreased luciferase activity of the *CA9* 3′UTR reporter with the C allele on rs1048638. However, luciferase activity of the *CA9* 3′UTR reporter carrying the C-to-A substitution of rs1048638 was not inhibited by miR-34a. These results were further supported by estimating the best minimal free energy (MFE) duplexes of the miR-34a and *CA9* 3′UTRs. The A allele of rs1048638 led to a far worse energy of −12.9 kcal/mole than did the C allele (−17.4 kcal/mole) with miR-34a hybridization (Fig. 2C). To confirm the importance of the rs1048638 polymorphism to the post-transcriptional regulation of CA9 expression by miR-34a, we examined rs1048638 genotypes of six HCC cell lines (Huh1, Huh6, Huh7, Hep3B, Mahlavu, and PLC5). Among the cell lines examined, only PLC5 carried rs1048638-CA (data not shown). Interestingly, high miR-34a-expressing cells, including Huh1 and Huh6, had low CA9 protein expression, while the lowest miR-34a-expressing cell, Mahlavu, had the highest CA9 expression (Fig. 2D). Furthermore, when we transfected miR-34a mimics into Huh7 and PLC5, CA9 mRNA and protein levels decreased in Huh7 cells but not in PLC5 cells (Fig. 2E). Taken together, these results suggest a regulatory route between miR-34a and CA9 expression, and the rs1048638 polymorphism determines the response to miR-34a targeting.

### miR-34a and the CA9 axis regulated cell proliferation and mobility of HCC cells

**Figure 3:**
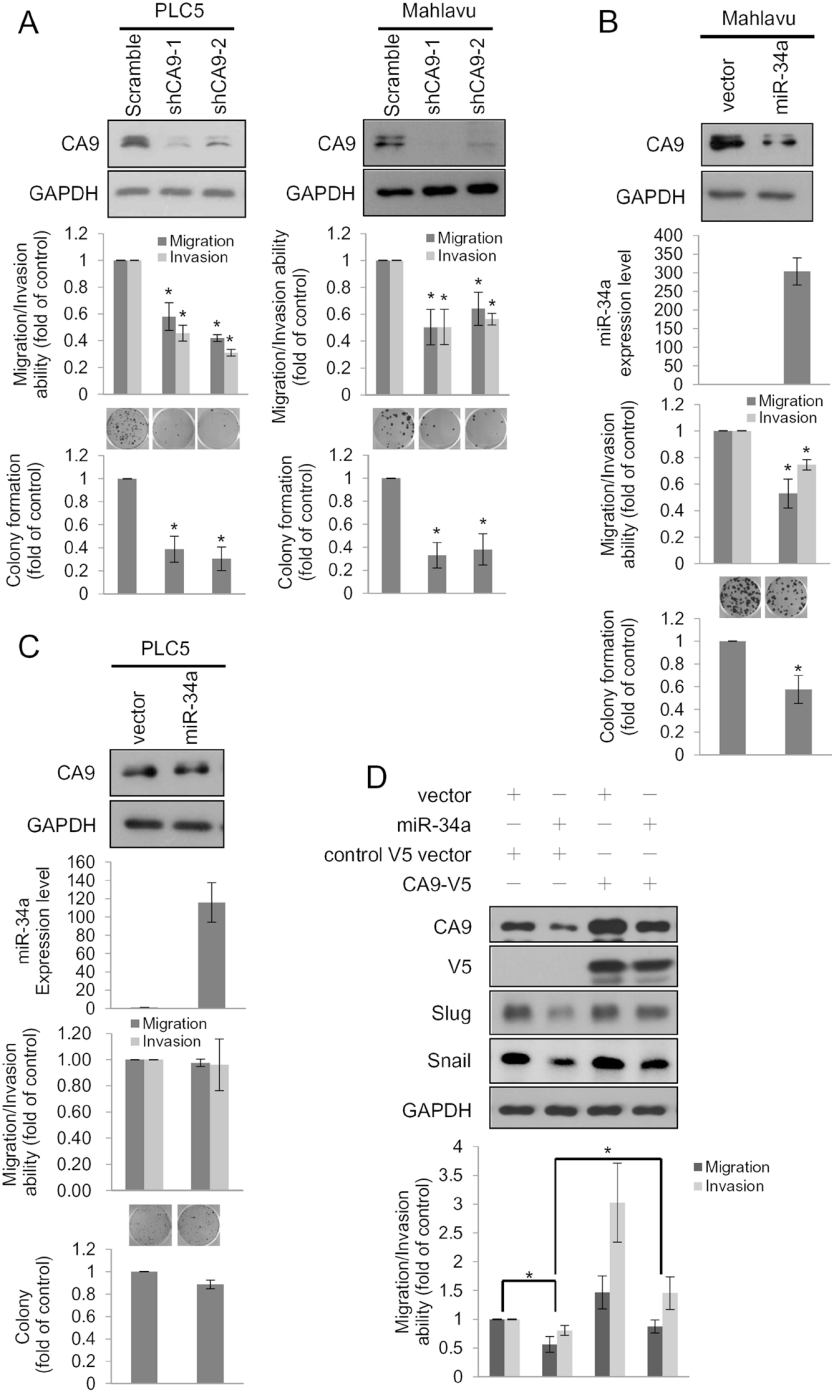
The miR-34a/CA9 axis regulates cell proliferation and mobility of hepatocellular carcinoma (HCC). (**A**) Migration, invasion, and colony formation assays were conducted after infection with a lentivirus carrying CA9 shRNAs or scrambled shRNA in PLC5 and Mahlavu cells. Upper panels: Western blot analysis of CA9. Middle panels: Migration/invasion abilities of CA9 knockdown cells. Lower panel: Colony formation abilities of CA9 knockdown cells. Representative micrographs of colony formation assays shown above the plot. (**B**,**C**) Migration, invasion, and colony formation assays were conducted after transfection of miR-34a or a control plasmid in Mahlavu and PLC5 cells. miR-34a and CA9 expression levels were examined by Western blotting and a real-time PCR, respectively, as shown in the upper two panels. Middle panels: Migration/invasion abilities of miR-34a-overexpressing cells. Lower panel: Colony formation abilities of miR-34a-overexpressing cells. Representative micrographs of colony formation assays shown above the plot. (**D**) Control and miR-34a-overexpressing Mahlavu cells were infected with lentiviruses carrying either control or CA9-V5 for 48 h and subjected to transwell migration and invasion assays. Migrated/invaded cells are presented as multiples of the control. Three independent replicates were performed for each experiment. Error bars, ±S.D. *p < 0.05 by two-sided Student’s *t*-test.

In addition to its crucial role in intracellular pH maintenance, the activity of CA9 also stimulates aggressive phenotypes of different cancer cells^18, 19^. However, the functional roles of CA9 in HCC cells remain largely unknown. We next examined whether CA9 expression affected cell proliferation and invasion of HCC. As expected, CA9 knockdown with two specific shRNAs significantly suppressed cell migration, invasion, and colony formation abilities of both PLC5 and Mahlavu cells (Fig. 3A). On the contrary, miR-34a was reported to inhibit cell proliferation, migration, and invasion of HCC cells^20, 21^. Consistently, miR-34a overexpression in Mahlavu cells, carrying rs1048638-CC, decreased cell migration, invasion, and colony formation abilities (Fig. 3B). However, miR-34a overexpression in PLC5, which carries rs1048638-CA, did not alter these cellular functions (Fig. 3C). We also observed a rescue effect of CA9 overexpression on the cell migration and invasion of miR-34a-overexpressing Mahlavu cells, further confirming the importance of the miR-34a/CA9 axis in regulating cell mobility of HCC (Fig. 3D). Since CA9 and miR-34a were found to regulate cell migration and invasion through regulating the epithelial-mesenchymal transition (EMT)^22–24^, we also examined EMT markers in Mahlavu cells after CA9 knockdown or miR-34a overexpression. As shown in Supporting Fig. 1, snail, slug, twist, and vimentin decreased following CA9 depletion and miR-34a overexpression in Mahlavu cells. CA9 overexpression, on the contrary, rescued miR-34a-inhibited snail and slug expressions (Fig. 3D). Collectively, these results suggest that the miR-34a-CA9 axis not only controls cell proliferation and mobility but also regulates the EMT process in HCC cells.

### miR-34a and the CA9 axis regulated the *in vivo* metastasis of HCC cells

**Figure 4:**
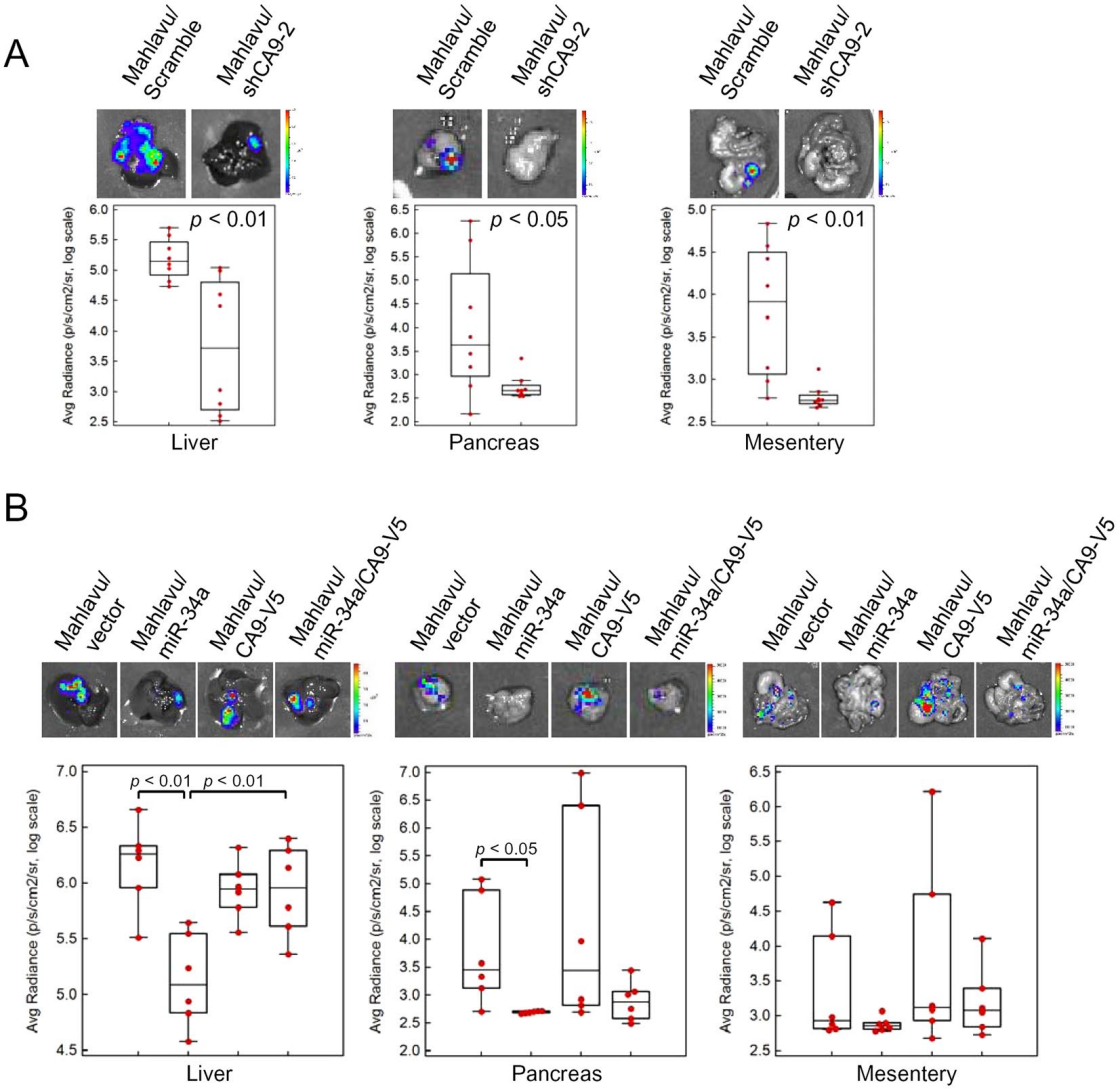
The miR-34a/CA9 axis regulates metastasis of hepatocellular carcinoma (HCC) *in vivo*. Mahlavu/Luc cells (10^6^) stably expressing CA9 shRNA-2 or miR-34a were orthotopically injected into NOD/SCID mice for 5 weeks. The tumor burden in the liver and metastases to the pancreas or mesenteries were estimated by luciferase activities. Representative photon images of the liver, pancreas, or mesentery were taken after sacrifice. (**A**) The tumor burden in the liver and metastases of CA9-knockdown cells. (**B**) The tumor burden in the liver and metastases of miR-34a- and/or CA9-V5 overexpressing cells. Statistical significance was assayed by two-sided Student’s t-test. *p* values are labeled in the plot.

As the cell mobility and EMT process are critical to the development of tumor metastasis^25^, we evaluated the effect of knocking down *CA9* on tumor growth and metastasis using xenograft models of luciferase-expressing Mahlavu cells. Cells were orthotopically injected into the left liver lobe of nonobese diabetic/severe combined immunodeficiency (NOD/SCID) mice. At 6 weeks after inoculation, primary tumors from CA9 knockdown cells showed a significant reduction in the luciferase signal compared to those of control-shRNA cells (Fig. 4A, left panel, *p* < 0.01, Student’s *t*-test). Furthermore, metastatic tumors were found in the pancreas and mesentery of mice injected with control cells, but loss of CA9 led to a significant decrease in metastatic dissemination (Fig. 4A, middle and right panels, *p* < 0.05 and <0.01, respectively). To determine the miR-34a/CA9 regulatory axis in HCC metastasis, Mahlavu cells with control, miR-34a overexpression, CA9 overexpression, or miR-34a/CA9 co-overexpression were tested with the same orthotopic injection model using NOD/SCID mice. Overexpression of miR-34a led to a significant decrease in the primary tumor size in the liver and CA9 co-overexpression reversed this effect (Fig. 4B, left panel, *p* < 0.01, Student’s *t*-test). Metastatic tumors were also barely detected in the pancreas and mesentery of mice injected with miR-34a-overexpressing cells compare with control cells (Fig. 4B, middle and right panels, *p* < 0.05 and =0.19, respectively), while CA9 co-overexpression slightly but not significantly increased pancreas and mesentery metastases. Taken together, these *in vivo* data provide further support that miR-34a and CA9 have functions in regulating tumor growth and metastasis of HCC cells.

### CA9 expression levels were correlated with miR-34a levels and rs1048638 genotypes in HCC patients

**Figure 5:**
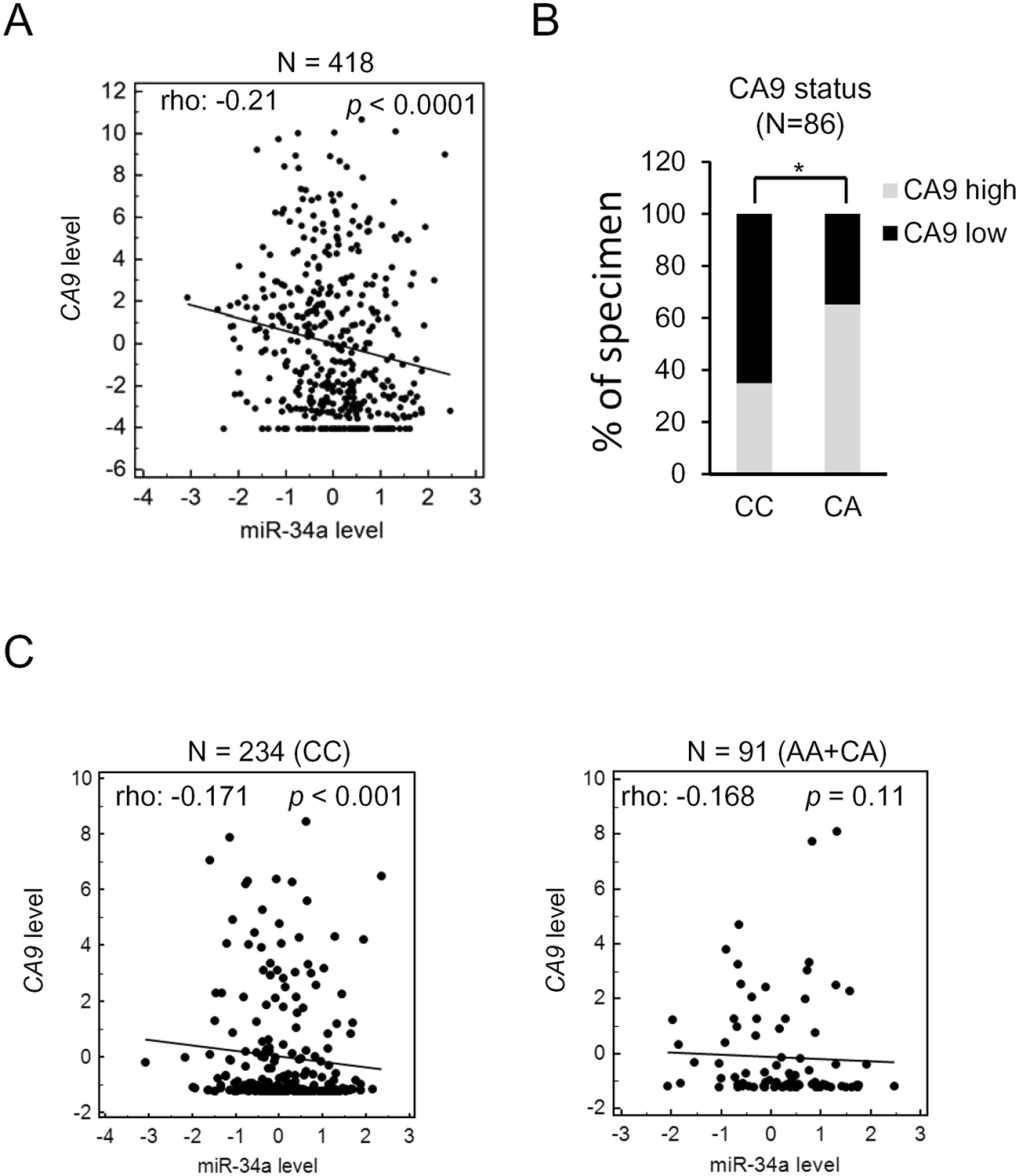
Association of rs1048638 with CA9 expression in hepatocellular carcinoma (HCC) patients. (**A**) RNA expression scatter diagrams of CA9 mRNA versus miR-34a. Black dots represent expression levels of both genes from specimens in TCGA HCC dataset; the regression line is shown on the plot. (**B**) Correlations between CA9 mRNA levels and rs1048638 genotypes in 86 HCC specimens. *p < 0.05 by Chi-squared test. (**C**) CA9 mRNA versus miR-34a expression scatter diagrams of specimens from rs1048638-CC or rs1048638-AA/CA cohorts. Statistical significance of (**A**) and (**C**) was assayed by Spearman’s rank correlation test. *p* values are labeled in the plot.

To evaluate the clinical correlations and importance of the rs1048638 polymorphism and miR-34a-CA9 regulation, we analyzed expression levels of miR-34a and CA9 in the TCGA HCC cohort, which is composed of 418 specimens. The correlation rho of *CA9* and miR-34a expression level was −0.21 (*p* < 0.0001, Spearman’s rank correlation test), suggesting a negative correlation between them (Fig. 5A). Furthermore, we compared CA9 expression levels in specimens with different CA9 genotypes to confirm their correlation. Our analysis showed that specimens with rs1048638-CC had higher CA9 expression, while specimens with rs1048638-CA had lower CA9 expression (Fig. 5B). To further evaluate the importance of the rs1048638 polymorphism on the CA9 targeting efficiency of miR-34a, we next analyzed the rs1048638 genotype of TCGA HCC cohort. We found that 234 specimens were rs1048638-CC, 82 specimens were rs1048638-CA, and nine specimens were rs1048638-AA. When comparing the correlation between miR-34a and CA9 expression, we observed a significant reverse correlation between them in specimens with rs1048638-CC (*p* < 0.001, Spearman’s rank correlation test). On the contrary, miR-34a expression was not correlated with CA9 expression in specimens with rs1048638-CA or -AA (*p* = 0.11, Spearman’s rank correlation test). Collectively, the above clinical analysis suggests a pivotal role of the *CA9* rs1048638 genotype in miR-34a-regulated CA9 expression in HCC.

## Discussion

CA9 is the most widely expressed gene in response to hypoxia. Its crucial role in intracellular pH maintenance represents the means by which cancer cells adapt to the toxic conditions of the extracellular milieu^26^. CA9 being expressed in many types of tumors indicates its relevance as a general marker of tumor hypoxia^11, 27^. Despite the molecular and cellular functions of CA9 being well-characterized, the impacts of its gene polymorphism in cancer incidence and progression are not fully understand. In this study, we identified a single SNP, rs1048638, in the 3′UTR of *CA9* that significantly increases HCC risk and was correlated with poor outcomes in HCC patients. Patients carrying the A allele at rs1048638 were associated with an adverse disease status, represented by a larger tumor size, a higher vascular invasion level, and more patients at a late pathological stage. Notably, *CA9* rs1048638-CA was associated with poor survival in uni- and multivariate analyses. In this regard, the *CA9* polymorphism at rs1048638 may represent a novel genetic risk factor and prognostic marker of HCC.

The expression of CA9 in normal tissues has a limited distribution in the gastrointestinal tract epithelium, ovarian coelomic epithelium, pancreatic ductal cells, hair follicle cells, and fetal rete testes^28, 29^. In contrast, CA9 is expressed in a variety of cancer tissues, including malignancies of the brain, head/neck, lung, breast, cervix uteri, kidney, and colon/rectum^30, 31^. The diagnostic value of CA9 can be traced to 1986, when a monoclonal antibody against G250, later revealing 100% identity to CA9, exhibited tumor-specific expression in many types of cancer^13, 32^. Interestingly, the level of CA9 expression is also associated with staging and survival prognosis in several types of human tumors. Higher levels of CA9 expression are associated with poor clinical outcomes in cervical, rectal, breast, lung, and brain tumors^33–35^. However, there are also reports suggesting that low levels of CA9 expression indicate a poor prognosis in cancers such as renal cell carcinoma and cholangiocarcinoma^36, 37^. Although the discrepancy might be related to different cutoff values proposed to discriminate between high and low expressions of CA9, it also implies that the CA9 expression level is not an unbiased prognostic marker. Besides, CA9 expression is highly regulated by hypoxia and results in the rather heterogeneous expression pattern of CA9 in tumors, rendering the measurement of CA9 expression more difficult^38^. Our previous studies of oral carcinoma suggested that the *CA9* polymorphism at rs2071676 was correlated with lymph node metastasis^15^. People with at least one A allele of CA9 rs1048638 had an increased risk of invasive urothelial cell carcinoma^16^. The synonymous C allele variant of rs12553173 was associated with improved overall survival in metastatic renal cell carcinoma^14^. We herein identified the *CA9* rs1048638 polymorphism as an independent prognostic factor in HCC. These lines of evidence suggest that CA9 SNPs may be suitable for predicting the risk and prognosis of cancers. As the cost and throughput of genotyping and DNA sequencing have rapidly improved in the past years, detecting the CA9 polymorphism may represent as a better strategy than assessing CA9 expression in the era of personalized medicine.

In the current study, frequencies of the CA genotype of rs1048638 were 18.3% (57/312) and 26.7% (23/86) in two Taiwanese HCC cohorts, comparable to the frequency estimated from TCGA HCC cohort (25%, 82/325). However, the AA genotype of rs1048638 was only present in TCGA HCC cohort at a frequency of 2.8% (9/325). This discrepancy may have been due to different prevalence rates of the “A” allele of rs168438 in eastern and western populations or the limited number of enrolled patients. However, since the AA genotype of rs1048638 was detected in 0.9% (4/462) of oral carcinoma cases and 1.4% (3/221) of urothelial cell carcinoma cases in our previous studies^15, 16^, the prevalence rate of the “A” allele of rs1048638 may be similar or slightly lower in eastern populations.

Several studies have demonstrated that CA9 overexpression promote tumor metastasis in animal models^19, 39^. However, we did not observed significant increase of pancreas and mesentery metastasis after CA9 overexpression in our orthotopic xenograft model. The discrepancy may came from the cell model we used in this study. Among HCC cell lines we surveyed for CA9 expression, Mahlavu cells expressed the highest level of CA9 (Fig. 2D). Forced expression of CA9 may not further increased the *in vivo* metastatic effects of endogenous CA9 protein due to the saturation of total CA9 protein in the cell. Although CA9 overexpression indeed increased migration and invasion abilities of Mahlavu cells *in vitro* (Fig. 3D), this may simply reflected the complex of metastasis *in vivo*. Moreover, orthotropic implantation of patient-derived intact tumor tissue in immunocompromised mice was reported to closely mimic the clinical pattern of metastasis^40–42^. An alternative *in vivo* imaging strategy with the use of fluorescent proteins also allowed monitored metastasis in real time^43–46^. Application of these animal models and *in vivo* imaging strategy in our future work will helped us further understand the roles of CA9 during HCC metastasis.

CA9 expression and activity in cancer cells are tightly regulated at multiple levels, including DNA methylation, transcription, post-translational modification, and proteinase-mediated cleavage^18, 47, 48^. Above all, *CA9* is best known as a hypoxia-inducible factor (HIF)-1α target gene under hypoxic conditions. However, little is known about the miRNA-dependent regulation of *CA9* expression in cancer. We herein report that CA9 is targeted by miR-34a, and this results in decreases in both CA9 mRNA and protein levels. Interestingly, like CA9, miR-34a is also regulated under hypoxic conditions. Du *et al*. reported that hypoxia induces downregulation of miR-34a expression and thus promotes the EMT^49^. Similarly, we also observed that several mesenchymal markers were suppressed by miR-34a or CA9 short hairpin (sh)RNAs. However, miR-34a is not directly regulated by HIF-1α. A recent report demonstrated that Snail2 suppresses miR-34a expression in hypoxia-induced mammospheres. Consistent with our findings, they also suggested that CA9 is a miR-34a target^50^. Recently, it was reported that CA9 in some types of cancer was predominantly regulated by epigenetic events, such as CpG methylation, rather than by hypoxia^47^. The roles of miR-34a-dependent CA9 regulation, as another level of epigenetic regulation, in cancer under hypoxia or normoxia are still worth investigating in the future. Furthermore, in contrast to CA9, miR-34a is downregulated in multiple types of cancer^51^. We also observed a reverse correlation between miR-34a and CA9 levels in HCC specimens. Hence, these findings support an important role of miR-34a in CA9 expression, and thus highlight the importance of rs1048638 polymorphisms in controlling CA9 expression.

In summary, we identified an SNP in the *CA9* 3′UTR as a novel diagnostic and prognostic factor for HCC. The rs1048638-A genotype significantly affects miR-34a targeting and expression levels of CA9. The miR-34a-CA9 axis is important in controlling tumor growth and metastasis of HCC cells. This indicates that the genetic variation at rs1048638 of the CA9 3′UTR plays an important role in regulating CA9 expression and cancer progression of HCCs, which is a novel determinant and target for HCC metastasis and prognosis. This provides further evidence for the important role of CA9 in HCC progression and new insights into the regulatory mechanism governing CA9 expression, which may be a novel therapeutic target for HCC.

## Materials and Methods

### Subjects and specimen collection

This study was approved by the institutional review board of Chung Shan Medical University Hospital (CSMUH)(Taichung, Taiwan). Subjects, including 312 patients with HCC and 312 cancer-free controls, were recruited in this investigation from 2007 to 2015, and all participants provided informed written consent at enrollment. A diagnosis of HCC was histologically confirmed in all cases. During the same study period, ethnically matched individuals who had neither been diagnosed with HCC nor had a self-reported history of cancer at any site were enrolled as controls. The TNM classification of the American Joint Committee on Cancer (AJCC) was used for staging of hepatocellular carcinoma. Whole-blood specimens collected from controls and HCC patients were placed in tubes containing ethylenediaminetetraacetic acid (EDTA), immediately centrifuged, and stored at −80 °C. Tumor RNA samples of 86 HCC specimens for a survival analysis were provided by the Taiwan Liver Cancer Network (TLCN). The TLCN is funded by the National Science Council to provide researchers in Taiwan with primary liver cancer tissues and their associated clinical information. The use of the 86 HCC tissues in this study was approved by the TLCN User Committee. All experiments were performed in accordance with relevant guidelines and regulations of TLCN and CSMUH.

### Genomic DNA extraction

Genomic DNA was extracted using QIAamp DNA blood mini kits (Qiagen, Valencia, CA, USA) following the manufacturer’s instructions. We dissolved DNA in TE buffer (10 mM Tris and 1 mM EDTA; pH 7.8) and then quantified it by measuring the OD260. The final preparation was stored at −20 °C and was used to act as templates for the polymerase chain reaction (PCR).

### Real-time PCR

Allelic discrimination of the CA9 rs2071676, rs3829078, and rs1048638 allelic polymorphisms was assessed with an ABI StepOne™ Real-Time PCR System (Applied Biosystems, Foster City, CA, USA), and analyzed with Sequence Detection Systems (SDS) vers. 3.0 software (Applied Biosystems) using the TaqMan assay. The final volume for each reaction was 5 μL, containing 2.5 μL TaqMan Genotyping Master Mix, 0.125 μL TaqMan probe mix, and 10 ng genomic DNA. The real-time PCR included an initial denaturation step at 95 °C for 10 min, followed by 40 cycles at of 95 °C for 15 s and then at 60 °C for 1 min. In addition, 376del393 allelic polymorphisms were assessed with the PCR as described previously^15^.

### Characteristics of miRNA candidates

In this study, we predicted the targets using the web-based tool, RNAhybrid on BiBiServ2 (http://bibiserv.techfak.uni-bielefeld.de/rnahybrid). RNAhybrid determined the most energetically favorable hybridization patterns using the minimum free energy (MFE) of two RNA fragments of different lengths, i.e., long (3′-UTR of CA9) and short (mature miRNA sequences). The parameters used in the analysis were: the number of hits per target–3 nucleotides; maximum mismatch size–1 nucleotide; overhangs–2 nucleotides; and the MFE considered for each microRNA/target duplex was higher than −15 kcal/mole assessable on a perfect match between the mature miRNA and its target^52^.

### CA9 3′UTR luciferase reporter assays

CA9 3′UTR luciferase assay constructs containing the rs1048638 C or A allele were purchased from Genecopoeia (Rockwell, MD, USA). 293T cells were co-transfected with the miR-34a or miR-449a mimic or negative control, and a vector containing the CA9 3′UTR. The pRL-TK Renilla control vector (0.1 μg) (Promega, Madison, WI, USA) was also co-transfected as an internal control for transfection efficiency. GenMuteTM siRNA & DNA Transfection Reagent (SignaGen Laboratories, Ijamsville, MD, USA) was used for this transfection process according to the manufacturer’s instructions. Cells were harvested at 48 h after transfection and analyzed for luciferase activity using the Dual-Glo Luciferase Reporter Assay System (Promega).

### Cell culture

The Huh1, Huh6, and Huh7 HCC cell lines were obtained from the Health Science Research Resources Bank (Osaka, Japan; JCRB0403). The Hep3B and PLC5 cell lines were obtained from the American Type Culture Collection (Manassas, VA, USA). HCC cells were cultured in Dulbecco’s modified Eagle’s medium (DMEM; Life Technologies, Grand Island, NY, USA) supplemented with 10% fetal bovine serum (FBS; Life Technologies). All cell types were cultured at 37 °C in a humidified incubator containing 5% CO_2_. Cells in the log phase of growth were harvested by trypsinization for use in various assays and *in vivo* studies.

### Lentivirus production and infection

A lentiviral vector and its packaging vectors were transfected into 293T packaging cells by calcium phosphate transfection. Briefly, 293T cells were split (10^6^) into 10-cm^2^ dishes 1 day before transfection. Cells were then transfected with 10 μg of CA9 shRNA or an miR-34a-expressing plasmid together with 10 μg of pCMVΔR8.91 (packaging vector) and 1 μg of pMDG (envelope vector). After 6 h of incubation, the transfection medium was replaced with fresh culture medium. Forty-eight hours later, the lentivirus-containing medium was collected from the transfections and spun down at 1500 rpm for 5 min to pelletize the cell debris, the supernatant was filtered with a 0.45-μm filter, and target cells were infected with the fresh lentivirus-containing medium supplemented with 8 μg/ml polybrene for 24 h.

### Migration and invasion assay

The migration and invasion assay was performed as previously described^53^. About 2 × 10^5^ cells were plated into the top chamber onto a Matrigel-coated (for invasion assay) or non-coated (for migration assay) membrane and allowed to invade into the lower chamber for 24 h. Invaded cells were fixed and stained with 0.2% crystal violet. Stained cells were quantified by counting.

### Western blot analysis

A Western blot analysis was carried out as previously described^53^ using the following primary antibodies: anti-CA9 (GTX128428, GeneTex, Irvine, CA, USA), anti-GAPDH (10494-1-AP, Proteintech, Chicago, IL, USA), anti-vimentin (5741, Cell Signaling Technology), anti-Snail (3879, Cell Signaling Technology), anti-Slug (9585, Cell Signaling Technology), and anti-Twist (GTX100619, GeneTex).

### Animal studies

All animal work was done in accordance with a protocol approved by the National Taiwan University College of Medicine and National Taiwan University College of Public Health institutional animal care and use committees. Age-matched non-obese diabetic severe combined immunodeficient (SCID) male mice (6~8 weeks old) were used. Mahlavu cells (10^6^) stably expressing CA9 shRNA or miR-34a were resuspended in 20 μL of a 1:1 mixture of PBS and Growth Factor Reduced-Matrigel (BD Labware, Bedford, MA, USA) and orthotopically injected into one lobe of the liver. Mice were sacrificed 4 weeks later, and the liver, pancreas, and mesenterium were removed. Metastatic lesions were monitored and quantified using a noninvasive bioluminescence system (IVIS-Spectrum, PerkinElmer, Waltham, MA, USA). Consecutive sections were also made for every tissue block of the organs and stained with hematoxylin-eosin (H&E).

### Real-time quantitative RNase H2-dependent PCR (rhPCR)

To examine *CA9* rs1048638 genotypes in 86 TLCN HCC samples, we performed a real-time rhPCR assay using the CFX Connect Real-Time PCR detection system (BioRad, Hercules, CA, USA). RNA (at 1 μg) was reverse-transcribed with an iScriprt cDNA synthesis kit (BioRad, Hercules, CA, USA), and complementary (c)DNA obtained was used for the real-time quantitative PCR. The rhPCR assay primers were designed by beacon designer version 8.0 and synthesized by IDT (Coralville, IL, USA): forward primer: 5′-GTAACTGTCCTGTCCTrGCTCAA-3′, and reverse primer: 5′-TATAAATATTTATTTTAAAAAATTTCTTrUGCAGA-3′. The reaction mixture (20 μl) contained 2 μl of the cDNA template, 0.4 μl each of the primers (10 μM), 200 mU RNase H2 (IDT, Coralville, IL, USA), and iQ SYBR Green Supermix (BioRad, Hercules, CA, USA) amplified as follows: denaturation at 95 °C for 3 min, followed by 40 cycles at 95 °C for 10 s and 60 °C for 30 s. Direct detection of PCR products was monitored by measuring the fluorescence produced due to SYBR Green dye binding to dsDNA after every cycle.

### Variant calling of TCGA exome-seq data

The exome-sequenced liver hepatocellular carcinoma (LIHC) and matched normal samples generated by The Cancer Genome Atlas (TCGA) project were downloaded from CGhub (https://cghub.ucsc.edu/) as Binary Sequence Alignment Map (BAM) files. The genotype of rs1048638 in each LIHC sample was called using the Unified Genotyper tool of Genome Analysis Toolkit (GATK) with default settings^54^. If the read depth in a given sample for rs1048638 was less than six, no call was made; otherwise, if non-reference allele frequency was less than 20%, the call was “CC”; if the non-reference frequency was greater than 80%, the call was “AA”; if it was between 20% and 80%, the call was “CA”.

### Xenograft mouse model

Male mice were randomly divided into groups of six to eight mice per group. Mahlavu cells (5 × 10^5^ cells) with CA9 knockdown, CA9 overexpressed, miR-34a overexpressed, or their respective controls were orthotopically injected into left liver lobe of NOD/SCID mice (6–8 weeks old). Six weeks after implantation, the mice were sacrificed and tumor imaging in the liver, pancreas, and mesentery were performed by administration of luciferin (Biosynth, A.G., Switzerland) and bioluminescence technology (Xenogen IVIS-100 imaging system). Photons emitted from specific regions were quantified using Living Image® software (Xenogen Corporation). The use of animals for this study was approved by the National Taiwan University College of Medicine Institutional Animal Care and Use Committee.

### Statistical Analysis

A goodness-of-fit v2 test was used to evaluate Hardy-Weinberg equilibrium for the biallelic markers. Differences in demographic parameters between HCC patients and cancer-free controls were estimated using Fisher’s exact test or the Mann-Whitney U-test. The adjusted odds ratios (ORs; AORs) with their 95% confidence intervals (CIs) obtained by multiple logistic regression models after controlling for other covariates were used to assess the correlation of genotype frequencies with the risk of liver cancer plus clinical characteristics. The haplotype-based analysis was conducted using the Phase program 26. A *p* value of <0.05 was considered significant. Data were processed using SAS statistical software (vers. 9.1, 2005; SAS Institute, Cary, NC, USA).

## Electronic supplementary material

Supplementary Information
