# Supplementary material for: 3′UTR polymorphisms of carbonic anhydrase IX determine the miR-34a targeting efficiency and prognosis of hepatocellular carcinoma
Source: Sci Rep. 2017 Jun 30;7:4466. doi: 10.1038/s41598-017-04732-3 (PMC5493636; doi:10.1038/s41598-017-04732-3)
Supplement: Supplementary file 1 — Supplementary Information [file 41598_2017_4732_MOESM1_ESM.doc]

**3'UTR polymorphisms of carbonic anhydrase IX determine the miR-34a targeting efficiency and prognosis of hepatocellular carcinoma**

Kuo-Tai Hua, Yu-Fan Liu, Chia-Lang Hsu, Tsu-Yao Cheng, Ching-Yao Yang, Jeng-Shou Chang, Wei-Jiunn Lee, Michael Hsiao, Hsueh-Fen Juan, Ming-Hsien Chien, Shun-Fa Yang

**Table S1.** Distributions of demographic characteristics in 312 controls and 312 patients with hepatocellular carcinoma

| **Variable** | **Controls (*N*=312)** | **Patients (*N*=312)** | ***p* value** |
| --- | --- | --- | --- |
|  |  |  |  |
| **Age (years)** | **Mean ± S.D.** | **Mean** ± **S.D.** |  |
|  | 61.50 ± 11.45 | 62.79 ± 11.90 | 0.170 |
| **Gender** | ***n* (%)** | ***n* (%)** |  |
| Male | 223 (71.5%) | 220 (70.5%) |  |
| Female | 89 (28.5%) | 92 (29.5%) | 0.791 |
| **Stage** |  |  |  |
| I |  | 120 (38.5%) |  |
| II |  | 82 (26.3%) |  |
| III |  | 89 (28.5%) |  |
| IV |  | 21 (6.7%) |  |
| **Tumor T status** |  |  |  |
| ≤T2 |  | 206 (66.0%) |  |
| T2 |  | 106 (34.0%) |  |
| **Vascular invasion** |  |  |  |
| No |  | 259 (83.0%) |  |
| Yes |  | 53 (17.0%) |  |
| **Distant metastasis** |  |  |  |
| No |  | 294 (94.2%) |  |
| Yes |  | 18 (5.8%) |  |

**Table S2.** Distribution frequency of *CA9* genotypes in 312 controls and 312 patients with hepatocellular carcinoma

| **Variable** | **Controls (*N*=312) *n* (%)** | **Patients (*N*=312) *n* (%)** | **OR (95% CI)** | ***p* value** |
| --- | --- | --- | --- | --- |
| **rs2071676** |  |  |  |  |
| AA | 86 (27.6%) | 90 (28.8%) | 1.00 |  |
| AG | 150 (48.1%) | 158 (50.6%) | 1.007 (0.695~1.458) | 0.973 |
| GG | 76 (24.3%) | 64 (20.6%) | 0.805 (0.516~1.256) | 0.338 |
| AG+GG | 226 (72.4%) | 222 (71.2%) | 0.939 (0.662~1.330) | 0.722 |
| **rs3829078** |  |  |  |  |
| AA | 291 (93.3%) | 289 (92.6%) | 1.00 |  |
| AG | 21 (6.7%) | 23 (7.4%) | 1.103 (0.597~2.037) | 0.754 |
| GG | 0 (0%) | 0 (0%) | -- |  |
| AG+GG | 21 (6.7%) | 23 (7.4%) | 1.103 (0.597~2.037) | 0.754 |
| **rs1048638** |  |  |  |  |
| CC | 277 (88.8%) | 255 (81.7%) | 1.00 |  |
| CA | 35 (11.2%) | 57 (18.3%) | 1.769 (1.124~2.785) | **0.013*** |
| AA | 0 (0%) | 0 (%) | -- |  |
| CA+AA | 35 (11.2%) | 57 (18.3%) | 1.769 (1.124~2.785) | **0.013*** |
| **376del393** |  |  |  |  |
| INS/INS | 239 (76.6%) | 225 (72.1%) | 1.00 |  |
| INS/Del | 71 (22.8%) | 81 (26.0%) | 1.212 (0.840~1.749) | 0.305 |
| Del/Del | 2 (0.6%) | 6 (1.9%) | 3.187 (0.637~15.952) | 0.158 |
| INS/Del+ Del/Del | 73 (23.4%) | 87 (27.9%) | 1.266 (0.883~1.815) | 0.199 |

The odds ratios (ORs) and with their 95% confidence intervals (CIs) were estimated by logistic regression models.

**Table S3.** Clinical status and *CA9* *rs2071676* genotypic frequencies in 312 patients with hepatocellular carcinoma

| **Variable** | **AA (*N*=90)** | **AG + GG (*N*=222)** | **OR (95% CI)** | ***p* value** |
| --- | --- | --- | --- | --- |
| **Clinical Stage** |  |  |  |  |
| Stage I/II | 53 (58.9%) | 149 (67.1%) | 1.00 | 0.168 |
| Stage III/IV | 37 (41.1%) | 73 (32.9%) | 0.702 (0.424~1.163) |  |
| **Tumor size** |  |  |  |  |
| ≤T2 | 55 (61.1%) | 151 (68.0%) | 1.00 | 0.243 |
| T2 | 35 (38.9%) | 71 (32.0%) | 0.739 (0.444~1.229) |  |
| **Vascular invasion** |  |  |  |  |
| No | 74 (82.2%) | 185 (83.3%) | 1.00 | 0.813 |
| Yes | 16 (17.8%) | 37 (16.7%) | 0.925 (0.485~1.764) |  |
| **Distant metastasis** |  |  |  |  |
| No | 88 (97.8%) | 206 (92.8%) | 1.00 | 0.087 |
| Yes | 2 (2.2%) | 16 (7.2%) | 3.417 (0.769~15.179) |  |
| **Child-Pugh grade** |  |  |  |  |
| A | 70 (77.8%) | 168 (75.7%) | 1.00 | 0.692 |
| B or C | 20 (22.2%) | 54 (24.3%) | 1.125 (0.627~2.017) |  |
| **HBsAg** |  |  |  |  |
| Negative | 54 (60.0%) | 125 (56.3%) | 1.00 | 0.550 |
| Positive | 36 (40.0%) | 97 (43.7%) | 1.164 (0.707~1.916) |  |
| **Anti-HCV** |  |  |  |  |
| Negative | 44 (48.9%) | 119 (53.6%) | 1.00 | 0.450 |
| Positive | 46 (51.1%) | 103 (46.4%) | 0.828 (0.507~1.352) |  |
| **Liver cirrhosis** |  |  |  |  |
| Negative | 16 (17.8%) | 49 (22.1%) | 1.00 | 0.397 |
| Positive | 74 (82.2%) | 173 (77.9%) | 0.763 (0.408~1.428) |  |

T2: multiple tumor of >5 cm or tumor involving a major branch of the portal or hepatic veins.

HBsAg, surface antigen of the hepatitis B virus; HCV, hepatitis C virus; OR, odds ratio; CI, confidence interval.

**Table S4.** Clinical status and *CA9* *rs3829078* genotypic frequencies in 312 patients with hepatocellular carcinoma

| **Variable** | **AA (*N*=289)** | **AG + GG (*N*=23)** | **OR (95% CI)** | ***p* value** |
| --- | --- | --- | --- | --- |
| **Clinical Stage** |  |  |  |  |
| Stage I/II | 184 (63.7%) | 18 (78.3%) | 1.00 | 0.159 |
| Stage III/IV | 105 (36.3%) | 5 (21.7%) | 0.487 (0.176~1.349) |  |
| **Tumor size** |  |  |  |  |
| ≤T2 | 189 (65.4%) | 17 (73.9%) | 1.00 | 0.407 |
| T2 | 100 (34.6%) | 6 (26.1%) | 0.667 (0.255~1.745) |  |
| **Vascular invasion** |  |  |  |  |
| No | 241 (83.4%) | 18 (78.3%) | 1.00 | 0.528 |
| Yes | 48 (16.6%) | 5 (21.7%) | 1.395 (0.494~3.938) |  |
| **Distant metastasis** |  |  |  |  |
| No | 272 (94.1%) | 22 (95.7%) | 1.00 | 0.761 |
| Yes | 17 (5.9%) | 1 (4.3%) | 0.727 (0.092~5.724) |  |
| **Child-Pugh grade** |  |  |  |  |
| A | 222 (76.8%) | 16 (69.6%) | 1.00 | 0.431 |
| B or C | 67 (23.2%) | 7 (30.4%) | 1.450 (0.572~3.671) |  |
| **HBsAg** |  |  |  |  |
| Negative | 161 (55.7%) | 18 (78.3%) | 1.00 | **0.035*** |
| Positive | 128 (44.3%) | 5 (21.7%) | 0.349 (0.126~0.967) |  |
| **Anti-HCV** |  |  |  |  |
| Negative | 151 (52.2%) | 12 (52.2%) | 1.00 | 0.994 |
| Positive | 138 (47.8%) | 11 (47.8%) | 1.003 (0.429~2.347) |  |
| **Liver cirrhosis** |  |  |  |  |
| Negative | 60 (20.8%) | 5 (21.7%) | 1.00 | p=0.912 |
| Positive | 229 (79.2%) | 18 (78.3%) | 0.943 (0.336-2.644) |  |

T2: multiple tumor of >5 cm or tumor involving a major branch of the portal or hepatic veins.

HBsAg, surface antigen of the hepatitis B virus; HCV, hepatitis C virus; OR, odds ratio; CI, confidence interval.

**Table S5.** Clinical status and *CA9* *376del393* genotypic frequencies in 312 patients with hepatocellular carcinoma

| **Variable** | **INS/INS (*N*=225 )** | **INS/Del+ Del/Del (*N*=87)** | **OR (95% CI)** | ***p* value** |
| --- | --- | --- | --- | --- |
| **Clinical Stage** |  |  |  |  |
| Stage I/II | 141 (62.7%) | 61 (70.1%) | 1.00 | 0.217 |
| Stage III/IV | 84 (37.3%) | 26 (29.9%) | 0.715 (0.420~1.219) |  |
| **Tumor size** |  |  |  |  |
| ≤T2 | 144 (64.0%) | 62 (71.3%) | 1.00 | 0.224 |
| T2 | 81 (36.0%) | 25 (28.7%) | 0.717 (0.418~1.228) |  |
| **Vascular invasion** |  |  |  |  |
| No | 189 (84.0%) | 70 (80.5%) | 1.00 | 0.455 |
| Yes | 36 (16.0%) | 17 (19.5%) | 1.275 (0.673~2.415) |  |
| **Distant metastasis** |  |  |  |  |
| No | 213 (94.7%) | 81 (93.1%) | 1.00 | 0.595 |
| Yes | 12 (5.3%) | 6 (6.9%) | 1.315 (0.478~3.620) |  |
| **Child-Pugh grade** |  |  |  |  |
| A | 172 (76.4%) | 66 (75.9%) | 1.00 | 0.914 |
| B or C | 53 (23.6%) | 21 (24.1%) | 1.033 (0.578~1.844) |  |
| **HBsAg** |  |  |  |  |
| Negative | 131 (58.2%) | 48 (55.2%) | 1.00 | 0.625 |
| Positive | 94 (41.8%) | 39 (44.8%) | 1.132 (0.688~1.864) |  |
| **Anti-HCV** |  |  |  |  |
| Negative | 118 (52.4%) | 45 (51.7%) | 1.00 | 0.909 |
| Positive | 107 (47.6%) | 42 (48.3%) | 1.029 (0.627~1.689) |  |
| **Liver cirrhosis** |  |  |  |  |
| Negative | 48 (21.3%) | 17 (19.5%) | 1.00 | 0.727 |
| Positive | 177 (78.7%) | 70 (80.5%) | 1.117 (0.602~2.073) |  |

T2: multiple tumor of >5 cm or tumor involving a major branch of the portal or hepatic veins.

HBsAg, surface antigen of the hepatitis B virus; HCV, hepatitis C virus; OR, odds ratio; CI, confidence interval.


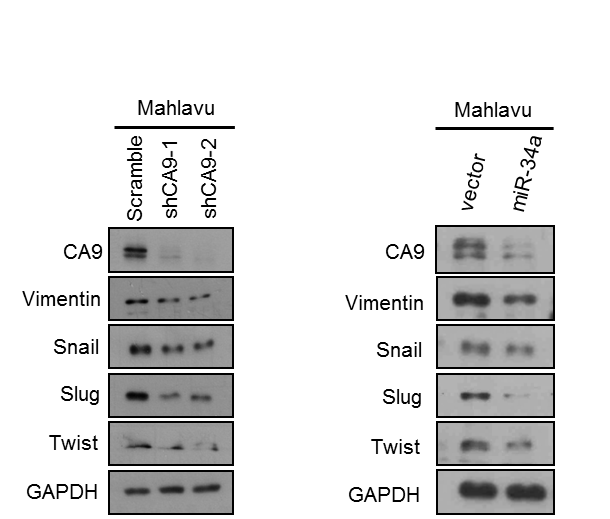


**Figure S1. The miR-34a/CA9 axis regulates epithelial-mesenchymal transition (EMT) marker expressions.** Western blot analysis of CA9 and EMT markers as indicated in CA9 knockdown or miR-34a-overexpressing Mahlavu cells. GAPDH was used as a loading control. Three independent replicates were performed in each experiment.
